# Deep Brain Stimulation as a Tool for Improving Cognitive Functioning in Alzheimer’s Dementia: A Systematic Review

**DOI:** 10.3389/fpsyt.2013.00159

**Published:** 2013-12-04

**Authors:** Katja Hardenacke, Elena Shubina, Christian Philipp Bührle, Alexandra Zapf, Doris Lenartz, Joachim Klosterkötter, Veerle Visser-Vandewalle, Jens Kuhn

**Affiliations:** ^1^Department of Psychiatry and Psychotherapy, University of Cologne, Cologne, Germany; ^2^Department of Stereotactic and Functional Neurosurgery, University of Cologne, Cologne, Germany

**Keywords:** deep brain stimulation, Alzheimer’s disease, dementia, cognition, animal models, memory, target selection

## Abstract

Deep brain stimulation (DBS) is an established, in selected cases therapeutically effective, non-lesional treatment method delivering current rectangular pulses into dysfunctional brain structures via chronically implanted stimulation electrodes. DBS is a recognized method applied in movement disorders and is increasingly evaluated as a possible therapeutic option for psychiatric diseases such as refractory obsessive-compulsive disorders, Gilles de la Tourette syndrome, major depression, and substance-related addiction. Latest research indicates that DBS may be a method for improving cognitive functions in Alzheimer’s dementia (AD). Translational data in healthy and AD animals appear to support this notion. Nevertheless, many aspects remain unclear, particularly with regard to the optimal target structure. The objective of this review is to present a systematic overview regarding published research on DBS and cognitive functioning in animal and human studies as well as to provide a systematic overview of the feasibility and efficacy of the treatment. We describe three studies investigating the effects of DBS in patients with dementia, using either the fornix or the nucleus basalis of Meynert (NBM) as a target. In total, we identified 25 animal studies with 10 brain structures being targeted: fornix, NBM, anterior caudate nucleus, dorsal striatum, anterior thalamic nucleus, midline thalamic nuclei, central thalamus, lateral hypothalamus, hippocampus (entorhinal cortex, perforant path), and amygdala. Considering the wide and diverse spectrum of targets, we add to this review a supposition about possible underlying mechanisms of operation and recommendations for further research.

## Introduction

Deep brain stimulation (DBS) is a technique affecting neuronal discharge patterns not only locally, i.e., within the target volume, but also in distant brain areas by conveying bioelectric impulses along projections originating in the target volume and terminating in remote structures. These projections may be uni- or bi-directional. DBS is based on (mostly) stereotactically guided insertion of stimulating electrodes into predefined target structures of the brain and the subsequent passing of electric pulses through these leads for influencing neuronal viz., bioelectrical activity there. Since its introduction in the late 1980s, chronic DBS has substantially expanded therapeutic options in movement disorders such as Parkinson’s disease, essential tremor, and dystonia (1, 2). Some studies have shown certain encouraging results implying DBS as a potentially effective but still highly experimental treatment modality for mental disorders. Most prominent are the following: obsessive-compulsive disorder [OCD; with FDA-approval under the human device exemption and also CE-mark; Ref. (3–5)], Gilles de la Tourette syndrome (6, 7), major depression (8, 9), or substance-related addiction (10). Based on apparently beneficial outcomes and in view of the largely favorable actions of DBS in addressing Parkinson’s disease, it is obvious to also discuss DBS in the context of Alzheimer’s dementia (AD). Both clinical entities are neurodegenerative disorders. Realizing that – despite great efforts – a satisfactory pharmacotherapeutic breakthrough using current strategies is still lacking, recent reports have shown first promising preliminary results of DBS as a therapeutic tool in patients suffering from AD, which prompted the idea of further investigating this approach. This is supported by the fact that a few translational studies (11) and other basic research activities (12, 13) also may hint to a potentially neuromodulative effect of DBS on cognition (even) in dementia. However, in all these studies different target structures for DBS have been used. The objective of this review is to give an overview about the currently emerging knowledge from research with regard to the usefulness of DBS in AD.

## Methods

According to the objective of our review we searched the PubMed^®^ database for relevant publications during the last decade (last update on 2013 May 21). By using various search terms like “dementia,” “cognition,” “memory,” and “neurogenesis” in combination with DBS a total of 558 results was obtained. We screened the abstracts and included investigations, if original data were presented, the manuscript had been published in English and referred to human or animal research *in vivo*. However, the most important selection criterion was that the studies explicitly reported the outcome of DBS treatment with the aim to modify cognitive accomplishment in demented patients. Thus, reports were eliminated in which the level of cognitive functionality was only assessed as a secondary outcome parameter, e.g., for excluding untoward effects. With investigations in animal models, the search criteria were extended to studies analyzing healthy animals for drawing long-term conclusions about the value of DBS for alleviating cognitive deficits. After screening all relevant studies and excluding those papers not fulfilling the inclusion criteria, we evaluated 28 [3 human (see Table 1); 25 animal studies] studies in further detail. These will be discussed below. Additionally, the reference lists of the publications were screened. By doing so, five further investigations attracted our attention [see Table 2; Ref. (12–16)]. Since these studies do not comply with our inclusion criteria, they were not taken into further consideration. First, we discuss findings from human case reports and clinical investigations. Secondly, we focus on animal findings, especially taking into account underlying hypotheses and conceivable targets. The studies are categorized according to their target structures.

**Table 1:** **Studies in man investigating the effects of DBS primarily addressing cognitive performance in patients suffering from dementia**.

Reference	Target structure	Patient population	Major outcome	Side effects	Stimulation parameter	Study duration
Laxton et al. (17)	Fornix	*N* = 6 mild to moderate AD	Rate of cognitive decline was diminished after DBS in these patients (assessed with ADAS-cog and MMSE), sustained changes in glucose metabolism	Sensation of warmth, flushing, and sweating, increases in heart rate and blood pressure	3.0–3.5 V 130 Hz 90 μs	52 months
Fontaine et al. (18)	Fornix	*N* = 1 mild to moderate AD	Memory scores stabilized and mesiotemporal lobe metabolism increased	No	2.5 V 130 Hz 210 μs	52 months
Freund et al. (19)	Nucleus basalis of Meynert (NBM)	*N* = 1 Parkinson’s dementia	Improvements in various aspects of cognitive capacity following NBM stimulation	No	1 V 20 Hz 120 μs	29 weeks

**Table 2:** **Studies in humans, which attract our attention and seemed to be relevant although not meeting our inclusion criteria (therefore they are at least listed in the following table)**.

Reference	Target structure	Patient population	Major outcome	Side effects	Stimulation parameter	Study duration
Fell et al. (12)	Rhinal cortex (RC), hippocampus (H)	*N* = 11 epilepsy	Linear effect of stimulation condition on memory performance (trend). Best performance in the in-phase condition, followed by the sham condition and the anti-phase condition	No	In mV range 40 Hz n.a.	Anti-phase (RC vs. H)/in-phase (RC and H vs. mastoid electrode)/sham comparison
Hamani et al. (14)	Fornix	*N* = 1 obesitas	Enhancement of autobiographic memory	>7 V: unpleasant warming sensation, facial hyperemia, and sweating	3 V 130 Hz 60 μs	3 weeks
Koubeissi et al. (16)	Fornix	*N* = 11 intractable epilepsy	MMSE scores showed an increase during stimulation compared to the baseline MMSE scores	No	8 V 5 Hz 0.2 μs	4 h
Stefani et al. (15)	Pedunculopontine tegmental nucleus (PPTg)	*N* = 6 morbus Parkinson	Increase of regional cerebral glucose metabolism (rCMRgI). Significant improvement of different cognitive functions	No	2.4 V 25 Hz 60 μs	52 months
Suthana et al. (13)	Entorhinal cortex (EC)	*N* = 7 pharmaco- resistant epilepsy	Enhancement of spatial memory performance. Resetting of the theta rhythm (EEG)	No	Up to 3.0 V 50 Hz 300 μs	ON/OFF comparison

## Human Studies

At the time of this writing, studies about the impact of DBS on memory and cognition in dementia are still quite rare. There is one case report [nucleus basalis of Meynert (NBM); Ref. (19)] and two feasibility studies [2× fornix; Ref. (17, 18)] investigating the effects of DBS on cognitive functions in Parkinson’s and AD respectively.

### Fornix stimulation

In 2008, a single case report on the effect of hypothalamic/fornix DBS for the treatment of obesity was published by Hamani et al. (14). A 50-year-old man was exposed to bilateral (ventral) chronic hypothalamic/fornix DBS for 3 weeks. However, the primary therapeutic objective of counteracting morbid obesity was a failure. Nonetheless, remarkable stimulation effects upon the modification of memory processes were observed incidentally. During intraoperative acute stimulation (pulse amplitude: 3.0 V, pulse width: 60 μs, and frequency 130 Hz), the patient reported sudden “dejà vu” – feelings, e.g., seeing himself in a situation 20 years ago. This artificially evoked autobiographical memory became more vivid when stimulation intensity was increased from 3.0 to 5.0 V. With increasing stimulation voltage still further (≥7 V), markedly stronger adverse effects, including the perception of an unpleasant generalized warming sensation, followed by facial hyperemia and sweating, were experienced. Neuropsychological testing at 2.8 V demonstrated an increase in recollection assessments at baseline before surgery as well as 3 and 52 weeks after continuous bilateral stimulation in a recognition task. This may suggest hypothalamic involvement in memory function. The authors concluded that hypothalamic/fornix DBS apparently influences bioelectric activity in limbic areas, thus exerting hippocampus-dependent memory-enhancing effects.

Based on that case report, this group started an open-label phase-I study (17). Here, six patients with mild and early AD were treated with bilateral fornix stimulation for 12 months. Electrodes were placed 2 mm anterior and parallel to the vertical portion of the fornix. Two weeks after surgery, chronic stimulation was commenced (amplitude: 3.0–3.5 V; pulse width: 90 μs; frequency: 130 Hz). Like surgery itself, this was well tolerated. Stimulation settings and pharmacotherapy (cholinesterase inhibitors) remained constant for 12 months, while extensive clinical and neuropsychological assessments were done 1, 6, and 12 months post-surgery. Clinical evaluation – using the Alzheimer’s Disease Assessment Scale [ADAS-cog; Ref. (20)] as the primary outcome criterion – suggested some post-surgical improvement, particularly in verbal recollection. However, the results of the ADAS-cog were variable among individual patients. A decelerated progression of cognitive decline was claimed and seemed to be reflected by the scores of the Mini Mental Status Examination [MMSE; Ref. (21)] indicated by a rate of decrease of 2.8 points preceding DBS, compared to a rate of decline of 0.8 points after surgery. Sustained alterations in cerebral glucose metabolism were also observed in frontal-temporal-parietal-striatal-thalamic and frontal-temporal-parietal-occipital-hippocampal networks after 1 year of DBS. A higher baseline metabolism and shifts in glucose utilization after 1 year coincided with improved clinical outcomes in cognition, memory, and quality of life (22). EEG-based analysis, such as standardized low resolution electromagnetic tomography, sLORETA (23), 6 and 12 months after the implantation of the electrodes showed that DBS apparently promotes neural activity in memory circuits including the entorhinal and hippocampal area thus modulating the default mode network. This network is characterized by coherent neural oscillations below 0.1 Hz, being activated during passive task states such as remembering or daydreaming (24). Taken together, these results suggest that neural activity in memory-relevant circuitry (involving fornix-hippocampal pathways) might be altered by fornix DBS, especially in entorhinal and hippocampal brain regions. As an interesting byline concerning potential future studies, the authors proposed that there is a relationship between disease severity and efficiency of fornix DBS. Patients with better pre-stimulation cognitive functionality are more likely to benefit from DBS than are patients with advanced AD. This hypothesis was supported by the observation that higher preoperative glucose metabolism apparently correlated with improved clinical outcome as far as cognitive functions and quality of life measures are concerned (22). Along these lines, it has been speculated that in individuals with mild and early AD functional integrity of the fornix-hippocampus circuit might still be intact, thus increasing the chances for a beneficial response to DBS. The authors asserted that it is the dysfunctional default mode network that should be subjected to stimulation instead of a specific target region in order to stabilize cognitive functioning.

Although this investigation provides indications that fornix stimulation might have memory-enhancing effects, extreme caution should be exercised when interpreting these results as a consequence of the open-label design, the small number of patients, and the absence of a control group (25).

A further study focusing on the feasibility and safety of DBS in AD was recently published by Fontaine et al. (18). In this investigation, 110 patients with established AD or mild cognitive impairment (MCI) were screened; of these, nine patients met the inclusion criteria of being below the age of 70 and having only a mild cognitive deficit with impairments predominantly involving episodic memory. However, only one patient agreed to participate in this investigation and to undergo surgery. The patient was stimulated bilaterally in the hypothalamus, with the target being in close proximity to the fornix. The exact location of the electrode was 2 mm anterior and parallel to the vertical portion of the fornix within the hypothalamus. The stimulus amplitude was 2.5 V with a frequency of 130 Hz and a pulse width of 210 μs. No complications were reported, and surgery was well tolerated. Prior to DBS, deficits were identified particularly well in episodic memory. Clinical and neuropsychological assessments were done 3 months and 1 week before surgery, as well as 3, 6, and 12 months thereafter. Results showed that memory scores (e.g., MMSE, ADAS-cog) were stabile after 1 year of continuous stimulation by contrast to control conditions, but nevertheless remained well below pre-surgical assessments. A subjective feeling of improvement was expressed by the patient herself. No significant changes were observed regarding behavior or mood and nor regarding PET results 1 year after surgery. However, the glucose metabolism was slightly improved in medial temporal lobes, which is not typical for the spontaneous evolution of the disease. Nevertheless, the results should be interpreted with utmost reserve, since only one patient was studied.

### Nucleus basalis of meynert stimulation

A unique feature in the course of both AD and Parkinson’s dementia (PDD) is basal forebrain degeneration including the latter’s cholinergic projections to the cortex. Neurostimulation of ascending basal forebrain projections of the NBM may, therefore, represent a new strategy for enhancing the residual nucleus basalis output. The relevance of the cholinergic forebrain for brain plasticity has, for instance, been illustrated by the reshaping of auditory receptive fields during and after stimulation of the NBM in the adult brain (26).

Based on this notion [for further details, see Ref. (19, 27)] we implanted in a patient with PDD – additionally to the nucleus subthalamicus (STN) electrodes to treat the motor symptoms – bilateral electrodes into the NBM (laterodorsal portion of the intermediate part) and implemented low-frequency DBS (20 Hz), intending to evoke excitatory effects of the residual NBM output. DBS of the STN was done using standard 130 Hz stimulation for evoking beneficial motor effects. Neuropsychological assessments including memory evaluation were performed 1 week prior to the operation and after surgery using different combinations of stimulation configurations: STN stimulation only, combined STN and NBM stimulation, and STN stimulation with NBM sham stimulation. Apparently, cognitive functions remained largely unaffected by electrical stimulation of the STN, whereas cognitive functions improved markedly with additional bilateral stimulation of NBM. In particular, partial functions such as attention, concentration, alertness, drive, and spontaneity were noticeably enhanced and, as a consequence, an improvement in mood and enjoyment of former activities was observed in the patient. Additionally, ideational and ideomotor apraxic symptoms were positively influenced following NBM stimulation [Ref. (28); for more information]^1^. Electrostimulation of the STN combined with sham stimulation of the NBM resulted in a marked deterioration of cognitive functions. One day after restarting NBM stimulation, a significant improvement in cognitive functionality was observed, and memory scores returned to the performance levels seen in the beginning of the combined bilateral stimulation. Based on this case report, we initiated a phase-I investigation with six patients with mild to moderate AD who were treated with DBS of the NBM (unpublished data)^2^.

## Translational Studies

With research in humans, the currently available database is extremely small and limited in scope, since only very few AD patients have been systematically treated with DBS so far. In order to augment the available data on DBS in humans and its effectiveness regarding cognition as well as to further support the notion that DBS might be an effective method to augment cognitive functioning in dementia, we also included animal data into this review. A total of 25 studies focusing on DBS-induced influences on memory, cognition, and/or neurogenesis were scrutinized. *In vitro* studies were only included when they referred to behavioral observations. In animal experimentation, DBS was studied in 10 brain regions: fornix, NBM, anterior caudate nucleus, dorsal striatum (DS), anterior thalamic nucleus (AN), midline thalamic nuclei (MTN), central thalamus (CT), lateral hypothalamus (LH), hippocampus enthorinal cortex (EC, perforant path), and amygdala. In analogy to the human data, we subsequently categorized the animal studies according to their targets.

### Fornix stimulation

One way to create an experimental model for memory depletion is to mimic memory impairment and cholinergic deficit by injecting rats with scopolamine, a muscarinic acetylcholine (ACh) receptor antagonist, inducing a blockade of ACh receptors in the hippocampus (29). Such a model was used in rats for evaluating DBS effects in the forniceal region on memory function (30). All animals were injected with scopolamine prior to behavioral testing. The stimulated animals performed much better than the sham-stimulated group. The authors asserted that memory enhancement was achieved by driving the fornix activity both orthodromically and antidromically by stimulating large myelinated axons. These results are illustrating the importance of the connection between hippocampus and AN as well as the antagonizing DBS effect on scopolamine receptor blockade in the hippocampus. No DBS-induced side effects on motor activity or anxiety level were observed.

### Nucleus basalis magnocellularis/of meynert stimulation

Some effects of NBM DBS on memory acquisition, consolidation, and content retrieval were investigated in rats (31). Stimulation (1 Hz, 100 μA) was able to improve memory acquisition possibly by involvement of early memory encoding and/or maintenance. Several suppositions are put forth by the authors: DBS might have enhanced ACh release from NBM neurons that in turn favorably influenced neural plasticity mechanisms and/or increased attention and the state of readiness. Further, the amygdala, a structure that is relevant to aversively motivated learning, could have been activated, leading to modulation of memory consolidation in other brain regions. Besides, GABAergic projections to the cortex could have interplayed with NBM cholinergic neurons to produce cortical plasticity (31).

Once again, NBM stimulation-induced facilitation of memory acquisition was observed in rats (32). Unilateral stimulation (100 μA, 1 Hz) before behavioral training resulted in enhanced memory acquisition when compared to DBS outside the NBM or to unstimulated animals. Expression of c-Fos, an early gene protein product, was increased by NBM DBS bilaterally in prefrontal cortical areas (orbitofrontal, prelimbic, and infralimbic cortices) and some hippocampal subregions [dorsal CA (cornu ammonis region) and ventral dentate gyrus (DG)]. The authors suggested that DBS-induced memory improvement was likely due to enhanced neural activity of brain structures related to the memory system.

Hotta et al. observed an increase in extracellular neuron growth factor (NGF) in rats due to electrical stimulation (50 Hz, 200 μA) of the NBM as measured by an immunosorbent assay (33, 34). This increase was sustained for hours after stopping the stimulation and was probably the result of an enhanced NGF-mRNA expression. Nicotinic (mecamylamine), but not muscarinic (atropine) ACh receptor antagonists abolished this NGF response (34). The authors argued that DBS-induced NBM activation might help to maintain neuronal plasticity and might have a protective effect on the cerebral cortex as well. However, the enhancement of ipsilateral NGF release was observed in adult, but not in aged rats (34). The authors claimed that NGF release in adult rats was mediated by nicotinic cholinergic inputs from the NBM to the cortex, which is reduced or absent in aged rats.

### Nucleus caudatus stimulation

Stimulation of the anterior caudate in rhesus monkeys allegedly improved learning by enhancing the acquisition of selective visuomotor associations (35). Here, the monkeys had to associate images with a specific movement by trial and error. High frequency stimulation (HFS; 200 Hz, 200 μA) during correct responses improved the initial performance, whereas stimulation during failed trials only impaired the learning process. Stimulation of the rostral putamen or low frequency stimulation (LFS; 20 Hz) did not influence learning performance. The authors proposed a direct involvement of the caudate in augmenting selective visuomotor associations during learning, probably at reinforcement time.

### Dorsal striatum stimulation

An adverse effect of unilateral left DS DBS was observed on memory in rats (36). Stimulation did not affect learning performance, but seemed to impair procedural memory (defined by chosen strategies during the task). GABA (but neither 5-HT, 5-HIAA, dopamine, DOPAC, HVA nor glutamate) neurotransmitter level was increased 19 h after DBS bilaterally in the DS. It can be supposed, that this long-term or delayed DBS effect on striatal GABAergic system could lead to a stronger inhibition of neuronal and synaptic activity thereby explaining the impairment in procedural memory. Another given explanation could be a left-right functional imbalance of neural activity due to unilateral stimulation. As the GABA level was increased bilaterally, the authors speculated that left and right DS interact either via corticostriatal fibers and their antidromic activation or via substantia nigra and the thalamus (36).

### Anterior nucleus of the thalamus stimulation

The anterior nucleus of the thalamus (AN) is one target region for DBS in epilepsy (37). Because the AN is assumed to be part of the memory circuit, Hamani et al. addressed the question if unilateral AN DBS in rats modulates memory performance [130 or 20 Hz; Ref. (38)]. High-current (500 μA) DBS impaired memory acquisition, whereas neither consolidation nor retrieval was influenced.

Brain slices of previously stimulated (bilaterally, 130 Hz, 500 μA) rats showed increased c-Fos expression in the regions connected to AN (cingulate cortex, orbitofrontal cortex, and lateral EC), implying that AN DBS might influence structures at a distance from the target as well. According to the authors, this indicates that AN DBS not just inhibits AN cells but could still drive local axonal fibers and influence further structures like the hippocampal formation. In fact, *in vivo* electrophysiological recordings revealed a reduced firing rate of gyrus dentate cells after high-current stimulation [500 μA; Ref. (38)].

By contrast, other studies reported beneficial effects of AN DBS on memory and neurogenesis. These discrepancies might originate from different time frames and general conditions of stimulation, e.g., the widely differing stimulation parameters and unknown pulse shapes. The initial impairment of memory performance after stimulation might be just transient and would subside with possibly induced plastic changes, leading to memory improvement when training is conducted at longer intervals (39). In this context the observed neurogenesis induced by DBS in the following three studies (39–41) is quite interesting.

In a study of Toda et al. AN stimulation was performed bilaterally for 1 h using variable frequencies [10, 50, 130 Hz; 2.5 V; Ref. (40)]. Already 3 h after stimulation AN activity was increased in stimulated rats compared to controls (as revealed by zif268, an early growth response protein, immunostaining). Cell proliferation was observed (via BrdU, that incorporates into new generated DNA, immunostaining) several days after and was two- to threefold increased 3 and 5 days after DBS in granule cell layer and subgranular zone of DG. At long-term (31 days after DBS) 46% of the newly generated cells were characterized as mature neurons (NeuN-positive; neuronal marker), while 7% were astrocytes (GFAP-positive; astrocyte marker), indicating that a large percentage of DBS-induced newborn cells of the DG develops into mature neurons without significantly altering the proportion of differentiating cell types.

In addition, HFS restored the effect of corticosterone, a suppressor of hippocampal neurogenesis (39, 40) in rats. Already 1 day after corticosterone injection the number of newborn cells was reduced by about 68.3% compared to vehicle treatment (40). In stimulated animals, due to counteraction of DBS on corticosterone, the cell number was only reduced by 23.2%. Similar observations were made after longer periods (28 days) with only about a third reduction of newborn cells due to the counteracting DBS effect. Further, the long-term effect of this counteraction was evident on behavioral level as well since the corticosterone-dependent impaired performance was reversed by DBS (39). This effect was attributed by the authors to neuroplastic changes that are surmised to require a considerable time to become structurally and functionally effective. Consistent with this, an increased hippocampal proliferation was observed in stimulated animals (BrdU immunostaining). The long delay between stimulation and behavioral testing could have assured a more proceeded functional maturation of newborn DG cells and hence a better memory performance.

Similar findings as to neurogenesis were also reported for mice (41). Thereby, HFS (130 Hz) selectively increased symmetric divisions of amplifying neural progenitors (ANPs; by 81% over unstimulated animals), and not the division of quiescent neural progenitors. ANPs evolve into migrating postmitotic precursors that slowly mature into granule neurons of DG. Hence, their division may be the key step in regulating neurogenesis. After HFS the number of matured neurons in the granular layer was increased by 53% (vs. non-stimulation). Stimulation of the frontal associative area of the cortex did not reveal differences between LFS and HFS, indicating that only HFS of the limbic circuit is able to produce such changes. These findings apparently support the assertion of an excitation-driven neurogenesis and consequential improvement in memory performance. However, it is far from clear, whether electrostimulation actually is able to promote neurogenesis. Since the general design of such experiments is frequently rather unclear, and the immunohistochemical methods used are far from specific and therefore hardly suitable to prove a causal relationship between DBS and neogenesis of operational neurons, these results and assertions should be viewed with all due caution.

### Midline thalamic nuclei stimulation

Arrieta-Cruz et al. used a transgenic mouse model of AD (TgCRND8 mice, Tg, characterized by reduction in overall CA1 basal synaptic transmission) to evaluate the actions of DBS in MTN on short-term memory (42). Prior to DBS (25 Hz, 300 μA), the Tg mice revealed worse memory acquisition and short-term memory than the wild type (WT), whereas continuous DBS was able to improve the behavioral performance of the Tg mice. To study proper synaptic transmission prior to the onset of pathological amyloid deposition, hippocampal slices from young mice were used for electrophysiological recordings *in vitro*. For instance, Schaffer collateral projections from the CA3 region were stimulated (10–100 μA) to activate CA1 neurons [for details, see Ref. (42)]. Synaptic transmission and plasticity in the Tg group was reduced compared to controls. The authors concluded that a synaptic dysfunction in the hippocampus might subsequently lead to progressive memory and learning impairment. Whereas short-term potentiation in WT was observed already after stimulation trains at 50 and 100 Hz, Tg mice showed this effect just at 200 Hz, suggesting that neurons in Tg mice are less excitable. Increased neuronal activity in DG and CA1 region induced by stimulation was confirmed by FosB staining. In addition, stimulation-induced synaptic changes led to the increase of α-secretase activity in CA1 (42). α-Secretase cleaves the APP at Aβ peptide sites and subsequently inhibits the generation of Aβ peptides, which are associated with the development of AD. Hence, these findings suggest that HFS enhances synaptic plasticity and memory performance and might modulate Aβ accumulation in the brain in a mouse model of AD.

### Central thalamus (CT) stimulation

Unilateral HFS (100 Hz, 1.5 mA) of the central lateral (CL) nucleus of the anterior intralaminar thalamus in rats led to transsynaptic activation of CL neurons and upregulation of immediate-early genes (c-Fos and zif268) in the motor cortex, anterior cingulate cortex, caudate-putamen, and hippocampus (43). This was associated with a prompt and daily growing memory improvement. Furthermore, the motor activity was increased through DBS as well, indicating generalized arousal, which could be responsible for the increased cognitive performance.

Influences of DBS in bilateral rostral intralaminar thalamic nuclei were examined in rats (44). Application of low currents (0.01 mA) enhanced memory performance whereas higher currents (0.1 or 1 mA) impaired it, demonstrating an inverted-U relationship between thalamic activity and behavioral performance (44). The rostral intralaminar nucleus of the thalamus seems to play an important part particularly in retrieval and facilitating memory-guided responding which in turn could be enhanced by low current stimulation.

Improvement of behavioral performance was achieved by DBS (50 Hz, 200–500 μA) in the CT of two macaque monkeys (45). Although, according to the authors, this improvement was likely due to increased arousal state.

Shah et al. performed DBS (130 Hz, 1.8–8 mA) in the CT in a macaque monkey (46). The high variability in the results [increased (3/32), decreased (1/32), mixed (14/32), or no effect (14/32) on behavior] following DBS revealed a high complexity of CT stimulation on arousal, behavior, and cognition.

### Lateral hypothalamus and amygdala stimulation

Segura-Torres and colleagues investigated whether post-training intracranial self-stimulation (ICSS), a form of DBS in which animals are electrically stimulated when pressing a lever, may facilitate explicit memory in rats (47, 48). Rats were implanted unilaterally with an electrode at the LH, into the fibers of the medial forebrain bundle, and were taught to self-stimulate by pressing a lever in a Skinner box (50 Hz, 10–250 μA).

When declarative/relational learning was required, the ICSS group performed better than non-stimulated group. Thus, the ICSS could have enhanced (hippocampus dependent) declarative or relational learning (47). Further, with intense training, both groups performed similarly, whereas with little training the amplifying group performed better than the non-stimulated group implicating the former achieved a reliable level of explicit memory (48). The authors proposed that (post-training) ICSS is more effective when morphological and physiological changes involved in memory consolidation are not fully matured as in case of little training.

In another investigation, memory retrieval was facilitated only at long-term (24 h after) and not immediately after the ICSS, indicating a time dependent effect of ICSS on memory retrieval (49). However, there was no difference between the ICSS group and animals with intermediate training sessions, suggesting that the ICSS effect on learning could be the same as additional training. Whether the main effect of ICSS was proactive, retroactive, or both remained unclear. Nevertheless, a strong long-term facilitation of memory retrieval was suggested by the authors (49).

In another experiment, the rats completed a single five-trial session and subsequently were exposed to ICSS (50). The retention-level was higher in the ICSS group at a retention time of 24 h and especially 48 h (not at 72 h) compared to controls. No modulating effects on performance were observed after ICSS before training, thus the authors suggested a retroactive ICSS effect on the consolidation process of previous learning. Additionally, the long-term ICSS effect was tested 7 days after the 48 h retention session. The further behavioral improvement of ICSS animals was smaller than in previous sessions and also smaller than the improvement of the control group. Though, the overall performance of the ICSS group was better compared to controls, suggesting that the effect of the ICSS treatment was sustained (50). This finding indicates memory facilitation induced by post-training ICSS by affecting the consolidation of (brief) previous learning.

Segura-Torres and colleagues studied the effects of LH ICSS on hippocampal (51) and amygdalar (52) gene expression (90 min and/or 4.5 h after ICSS). c-Fos immunolabeling revealed increased activity in the amygdala, pyramidal layer (CA3), and granule cell layer (DGmb and DGlb). The hippocampal cell labeling varied in intensity, signifying that ICSS leads to activation of gene transcription in distinct cells (51). In spite of unilateral stimulation, the neurochemical effects were predominantly bilateral. In both stimulated regions, hippocampus and amygdala, increased expression of COX-2 gene which is related to neurite outgrowth and neurogenesis was observed (51, 52). Furthermore, genes related to protein folding (Hspa1a), neurite outgrowth and neurogenesis (Ret), neuroprotection (Hspa1a, Ret, Dnajb1), and anti-apoptosis (Hspa1a, Ret, Dnajb1, Fkbp5) were upregulated in both regions. Increased BDNF and Arc gene expression which is believed to be related to synaptic plasticity, was observed in the amygdala, but not examined in the hippocampus. Expression of even more genes was induced in the hippocampus by LH ICSS, which was not affected (e.g., Sgk, Pde1a) or not evaluated (e.g., Ubqln1) in the amygdala. In total, the expression of 62 early genes was influenced in the hippocampus by ICSS. Fifty-one percentage of the 37 expressed genes that encode proteins of known or presumed functions, may directly or indirectly promote learning and memory or neuroprotection (51). Recently, the research group additionally observed increased expression of early gene proteins, c-Fos and Nurr1 (transcription factor expressed in dopamine cells) due to stimulation (53). The changes in gene expression were predominantly ipsilateral [for details, see Ref. (53)]. The c-Fos enhancement (with and without conditioning) was observed in almost all parts of brain regions studied (amygdala, hippocampus, DS, lateral hippocampus). By contrast, no enhancement in Nurr1 expression was observed in the amygdala. This rising was measured 70 min, but not 48 h after stimulation suggesting a rapid and transient increase in expression. In some hippocampal regions, acquisition of behavioral testing alone was able to facilitate expression of these early genes. However, the combination with ICSS had a greater effect. In some regions it seemed like the test acquisition (+ICSS) could not additionally affect the gene expression in LH compared to ICSS alone. The authors concluded that c-Fos and Nurr1 mediate the facilitative effects of ICSS on memory consolidation. Further they proposed that the observed upregulation in genes involved in neurogenesis could indirectly facilitate learning and memory: the newly generated cells could be protected by products from upregulated genes related to neuroprotection, which could protect preexistent neurons as well. As an example, the authors took the gene Ubqln1 which is related to the regulation of protein degradation and may reduce protein aggregates and their toxicity. Such protein aggregates are known from AD and these findings give a possible approach to prevention of aggregation through DBS. Overall, these studies demonstrated that ICSS modulates hippocampal and amygdal activity and the regulation of gene expression, which differs between these regions. Thus, ICSS may facilitate learning and memory by enhancing cellular and molecular mechanisms related to neuroplasticity, neurogenesis, and neuroprotection (51, 52).

### Hippocampus, EC, and perforant path stimulation

Concerning neurogenesis, similar findings as with DBS in AN (39–41) were made by Stone et al. while investigating the outcome of acute uni- or bi-lateral stimulation (130 Hz, 0–500 μA) in the EC in mice (11). Activity in the granular cell layer of the middle and posterior regions of the ipsilateral DG increased following unilateral EC stimulation at low current density (50 μA, 30–120 min), inferred by c-Fos staining. The number of proliferating cells in the ipsilateral DG was transiently increased, especially in the granular cell layer of the middle and posterior regions of the ipsilateral DG. As in the study of Toda et al. maximum increase was observed about 3–5 days after stimulation. Increase in stimulation duration from 30 to 60 min was coincident with higher proliferation rates, whereas increasing stimulation duration to 120 min did not result in any gain. Stimulation increased the differentiation into neurons and had a modest pro-survival effect on existing nerve cells. Cell characteristics (rate of neuronal differentiation, localization, morphology, afferent and efferent connections, long-term survival) were comparable to nerve cells generated under control conditions. According to the authors these results imply that the new neurons integrated into hippocampal circuitry. The behavioral performance was improved when the animals had training 6 weeks – rather than 1 week – after DBS (unilateral and bilateral), when the new neurons were fully matured and integrated into the memory circuits, as proposed by the authors. The facilitated spatial learning and memory retrieval coincided with an increased number of activated neurons in stimulated animals compared to unstimulated controls (11). If the mice were pretreated with agents known to inhibit neurogenesis (e.g., the DNA-alkylating agent temozolomide), both proliferation as well as spatial memory performance were reduced in all mice, but to a higher extent in the unstimulated subgroup. According to the authors, this confirms that the improved cognitive effects originated, at least in part, as a result of stimulation-promoted hippocampal neurogenesis.

McCartney et al. demonstrated in rats that HFS (200 Hz) of the perforant path, a connection from the EC to all fields of the hippocampus formation, triggers theta phase resetting and the release of ACh, an alleged prerequisite for creating conditions favoring long-term potentiation [LTP; Ref. (40, 54)]. The authors speculate that HFS probably disrupted the circuits involved in encoding and retrieving stimuli information in the working memory task. Subsequently, theta phase resetting was believed to be associated with short-term encoding and retrieval and not only to long-term memory formation. Apparently, it can be speculated that theta resetting might synchronize incoming stimuli with hippocampal circuits with the aim of ensuring favorable conditions regarding the facilitation of encoding of the information into memory.

## Discussion

We reviewed the literature of the last decade and presented an overview of human and animal studies investigating the effects of DBS on cognitive functions with the primary aim to assess the potential of using DBS in the therapy of dementia. The available clinical evidence in human case studies comprises only a total of three investigations. The primary intent was to enhance cognitive functions in patients with dementia utilizing through DBS (17–19). [We identified five further potentially interesting studies dealing with the effects of DBS on several cognitive functions in other patient groups (13–16); because of not fulfilling our inclusion criteria they are only mentioned in Table 2.]

Considering the average low complication rate in a cohort of approximately 100,000 Parkinson patients treated with DBS globally (55, 56) DBS seems to be a feasible and safe treatment for patients with dementia. Independently of the target structure being stimulated, patients recovered quickly from surgery and only rarely experienced cognitive deterioration caused by the intervention.

Judging from an extremely small number of cases, the fornix is currently the target most frequently chosen for treating dementia (one phase-I study and one feasibility study). Generally, chronic stimulation was well tolerated, except for sensations of warmth, flushing, and sweating as well as increases in heart rate and blood pressure at higher stimulus voltages (>7 V) in two studies (14, 17). The neuropsychological results were highly variable both in validity and content, but might be interpreted with considerable reserve as indicating a stabilization of the disease progression after surgery, although there are no hard facts supporting this as yet. The stimulation parameters in both studies are similar with respect to voltage (2.5–3.5 V) and frequency (130 Hz). Pulse width varied between 60 and 90 μs. Although, taken at face value, these results may be seen as encouraging, they are still rather ambiguous. As a consequence, these observations may be spurious and should therefore be contemplated with all due caution. This is particularly true, when the episodic nature of the findings and the extremely small number of cases, defying proper statistical treatment, are factored in (*N* = 6 and *N* = 1). This is aggravated by the fact that only open-label designs were used and by the lack of a control groups. Supporting the idea of the fornix being an acceptable target structure, we gave an account of a study investigating the acute effects of DBS of the fornix on specific cognitive performances in patients with intractable epilepsy. This report alleged improved neuropsychological scores assessed with the MMSE during stimulation (16). However, the stimulation parameters were different compared to the fornix DBS studies in dementia, with higher voltages (8 V), LFS (5 Hz), and narrow pulse width (0.2 μs). They are therefore not comparable. Hence, the majority of the pivotal questions raised in this context still remain unanswered.

Support for such a tentative approach may be derived from a single animal study (30) which ascribed improved memory performance to fornix DBS (measured by means of the object location task) in rats, which had received an injection of scopolamine, a muscarinic ACh receptor antagonist, allegedly causing a blockade of ACh receptors in the hippocampus and thus mimicking some aspects of AD. Fornix DBS was reported to reverse the scopolamine action.

As to a further potential target structure, there is one investigation dealing with the effects of low-frequency (20 Hz) DBS in the NBM for treating a patient with PDD, stressing patient safety, and technical feasibility of this method and also auspicious results regarding memory and other cognitive realms, such as attention, visual processing, or practical symptoms (19). Data of a phase-I investigation with six patients with mild to moderate AD treated with DBS in the NBM are currently under review (see text footnote 2). In three animal studies (31, 33, 34) NBM DBS indicated an improvement in memory performance. It was claimed that, as a result of ACh release from the NBM, attention, and state of readiness are enhanced and neuroplastic mechanisms may be influenced positively. Additionally, NGF secretion was increased throughout the entire cortex, possibly mediated by its nicotinic cholinergic inputs from the NBM. The authors also alleged that memory improvement may be associated with an increased c-Fos expression in the stimulated brain regions; this claim, however, should be contested on the basis of possibly flawed reasoning. In a recent review of Gratwicke et al. the potential of the Nucleus basalis Meynert as a target for DBS in dementia was emphasized by characterizing the anatomy, intrinsic organization, and connectivity of the cholinergic nucleus. The authors believe that especially the afferent and efferent projections of the NBM, the 90% cholinergic neurons, and the early degeneration in AD make the Nucleus basalis Meynert a promising target structure in dementia (57).

Taking into account the results of both human and animal studies which are still rather scant and hard to interpret correctly, the NBM seems to be an auspicious target when it comes to future evaluations of its suitability as a structure to be stimulated for alleviating cognitive deficits in dementia.

Other potential DBS targets which might warrant evaluation in patients with dementia are the entorhinal cortex (13) and the pedunculopontine tegmental nucleus (15), because at least in some areas of cognition such as spatial orientation (EC), executive functions, and attention (PPTg), slight improvements appeared to occur in humans. Animal studies support the potential of the EC as a target for DBS. Stimulating the EC in mice, the neogenesis of neurons transiently increased and a modest pro-survival effect on existing cells could be observed (11).

Similarly, it was shown in rodents that electrical stimulation of the perforant pathway, a route connecting the entorhinal cortex to virtually all fields of the hippocampal formation, may trigger a theta phase resetting, thereby possibly creating favorable conditions for long-term potentiation. These results may confirm the hypothesis that resetting theta activity probably facilitates the potentiation and encoding of relevant incoming stimuli (54). There was no translational data available investigating the effects of stimulation of the PPTg.

In addition to the experimental investigations already alluded to, further translational studies were found in the literature which assessed effects on cognition after electrically stimulating brain regions for which data in humans are still lacking. The following structures are involved here: LH, central thalamus, amygdala, anterior nucleus of the thalamus, DS, nucleus caudatus, midline thalamic region, and perforant pathway of the hippocampus.

Lateral hypothalamus DBS was accomplished via ICSS, a method based on the activation of the reward system. Consequently, ICSS enhancement of memory and cognition may, however, just be the result of evoking unspecific neural arousal. However, such a facilitation was observed in a multitude of experiments (47–51, 53) and was, for instance, associated with enhanced expression of genes related to memory, cognition, neurogenesis, and neuroprotection. This would turn LH into an interesting target for DBS. Similar enhancement of gene expression was observed in a single study of amygdala ICSS (52). Nevertheless, further investigations are required, before any reasonable conclusions may be drawn.

The stimulation effects in CT were quite inconsistent and appeared to depend even more critically on the stimulation parameters compared with DBS in other targets (43–46). Here, the choice of stimulation parameters was demonstrated to be essential for discerning between improvement and impairment of cognition (44). A generalized and unspecific arousal is most likely responsible for improved memory performance, although CT DBS-induced activation of immediate-early genes could be a promising indication for a potential target structure which would merit further exploration.

Several animal studies claim that excitation-associated cell proliferation and hippocampal neurogenesis may be traced back to AN DBS, implicating the target as very interesting (39–41). Reported outcomes pertaining to influence general memory performance by AN stimulation are rather contradictory (38, 41), which probably is due to different stimulation parameters. This should be thoroughly clarified in further investigations, especially since in some studies flawed or inappropriate methodology, but also biased reasoning cannot be excluded.

Deep brain stimulation in the DS allegedly impaired memory performance in a single observation (36). This clearly does not suffice for a meaningful evaluation of this target structure and, consequentially, asks for further investigation.

Single translational studies reported on improved memory performance after DBS in the nucleus caudatus (35) and in the midline thalamic region (42), with the latter using an AD mouse model. Moreover, in the transgenic mouse, α-secretase activity in the CA1 region increased after DBS and thus might affect Aβ accumulation in AD. Accordingly, this effect should be observed in future related investigations.

Unfortunately, translational research of DBS is severely limited in many respects, making extrapolation of the results obtained and the conclusions drawn precarious if not epistemologically impossible when the situation in the much more complex and intricate human brain is concerned. This may be exemplified as follows: in contrast to clinical treatment, only acute and short-lasting stimulation (minutes to days) was used in most animal studies referred to here, likely resulting in decreased or even completely different effects. In many animal experiments, the investigation of the actions of neurostimulation was frequently done only a short time after the onset of the stimulation. It is evident that this time frame is much too short for detecting possible neuroplastic changes which require periods ranging from days to several months to develop and stabilize. It goes without saying that this holds especially in cases, where changes in the hard-wiring of neuronal circuitry are involved.

The animal experiments presented in this review were notorious for their extreme variability as far as stimulation parameters are concerned (pulse amplitude, duration, frequency, polarity, monopolar or bipolar, and shape, monophasic or biphasic). In addition, electrode geometry, material as well as area and shape of contacts are mostly unknown, resulting in ill-defined and vastly different current densities in the respective targets. Given the nearly infinite amount of possible permutations of the classical stimulus parameters, the odds for attaining comparable results in rats and humans are very small. Adding differences in electrode geometry, contact configuration, electrode material which may be polarizable or non-polarizable (Ag/AgCl_2_), the notion that – at the present stage of affairs – any results from rodent experimentation may be transferred to the situation in humans would verge on wishful thinking.

An equally severe conundrum lies with the fact that the effects of intracranial depth stimulation in experimental studies almost exclusively were observed in naïve animals not actually suffering from dementia, therefore lacking the structural damages as well as the pathophysiology typically underlying dementia. Accordingly, it would be a grave misconception, if an attempt was made to extrapolate experimental results obtained, for instance, in essentially healthy rodents to patients suffering from a chronically progressive neurodegenerative disease like AD (58). It also fundamentally confounds the concept of “translational research” that the majority of the investigations quoted here used adult but not aged animals which do not match the average age of the affected patient group.

Many transgenic and non-transgenic animal models with Aβ and/or tau pathology are used for the study of AD [for a review, see Ref. (59)]. Implementation of such models in DBS-related investigations may increase the information value (face, predictive, and construct) of this kind of experimentation, since more supporting evidence for possible mechanisms underlying the still elusive mode of action of DBS on memory in dementia might emerge.

Nevertheless, successfully translating data and conclusions drawn from phylogenetically rather primitive animals to humans without meticulous planning at all stages to most closely and faithfully model the situation obtaining in actual dementia patients appears to be an exercise in futility.

However, studies investigating the mode of action of DBS on cognition in healthy subjects would probably provide certain clues concerning its functionality in patients suffering from dementia. In conclusion, the investigated studies – human and animal research – indicated that the facilitating effect of DBS is most likely a markedly multi-factorial phenomenon comprising a host of vastly different mechanisms from nearly every field covered by the basic sciences. Apart from classical neurophysiology and neuroanatomy also genetics, neurochemistry, cybernetics, and non-linear dynamics including the theory of coupled oscillators, i.e., mathematical physics as well as linear and non-linear systems theory, are involved here.

Taking this into account, continuing clinical research efforts as well as animal experimentation addressing DBS efficiency in future treatment of dementias with a neurodegenerative basis such as Alzheimer’s disease appear to be worthwhile. Only the future will show whether this approach is a successful one.

## Author Contributions

Jens Kuhn, Katja Hardenacke, and Elena Shubina were responsible for the concept of the Review. Jens Kuhn, Katja Hardenacke, Elena Shubina, and Alexandra Zapf were responsible for the literature research. Jens Kuhn, Katja Hardenacke, Elena Shubina, Christian Philipp Bührle, Joachim Klosterkötter, and Veerle Visser-Vandewalle drafted the manuscript. All authors have critically reviewed content.

## Conflict of Interest Statement

Jens Kuhn has occasionally received honoraria from AstraZeneca, Lilly, Lundbeck, and Otsuka Pharma for lecturing at conferences and financial support to travel. Jens Kuhn received financial support for IIT-DBS studies from Medtronic AG (Meerbusch, Germany). Doris Lenartz reports having received financial assistance for travel to congresses from Medtronic AG. The other co-authors declare that the research was conducted in the absence of any commercial or financial relationships that could be construed as a potential conflict of interest.

## Footnotes

^1^http://onlinelibrary.wiley.com/doi/10.1002/mds.23141/suppinfo

^2^http://clinicaltrials.gov/ct2/show/NCT01094145?term$=$DBS$+$Alzheimer&rank$=$2; in review
